# Mechanism of Intermittent Deep Tillage and Different Depths Improving Crop Growth From the Perspective of Rhizosphere Soil Nutrients, Root System Architectures, Bacterial Communities, and Functional Profiles

**DOI:** 10.3389/fmicb.2021.759374

**Published:** 2022-01-10

**Authors:** Yabing Gu, Yongjun Liu, Jiaying Li, Mingfeng Cao, Zhenhua Wang, Juan Li, Delong Meng, Peijian Cao, Shuhui Duan, Mingfa Zhang, Ge Tan, Jing Xiong, Huaqun Yin, Zhicheng Zhou

**Affiliations:** ^1^School of Minerals Processing and Bioengineering, Central South University, Changsha, China; ^2^College of Agronomy, Hunan Agricultural University, Changsha, China; ^3^Tobacco Research Institute of Hunan Province, Changsha, China; ^4^Yongzhou Tobacco Company of Hunan Province, Yongzhou, China; ^5^Changde Tobacco Company of Hunan Province, Changde, China; ^6^Zhangjiajie Tobacco Company of Hunan Province, Zhangjiajie, China; ^7^China Tobacco Gene Research Center, Zhengzhou Tobacco Research Institute of CNTC, Zhengzhou, China; ^8^Xiangxizhou Tobacco Company of Hunan Province, Jishou, China

**Keywords:** rhizosphere bacterial community, intermittent deep tillage (IDT), root system architecture (RSA), sustainable agriculture, ecosystem function

## Abstract

Long-term conventional shallow tillage reduced soil quality and limited the agriculture development. Intermittent deep tillage could effectively promote agricultural production, through optimizing soil structure, underground ecology system, and soil fertility. However, the microecological mechanism of intermittent deep tillage promoting agriculture production has never been reported, and the effect of tillage depth on crop growth has not been explored in detail. In this study, three levels of intermittent deep tillage (30, 40, and 50 cm) treatments were conducted in an experimental field site with over 10 years of conventional shallow tillage (20 cm). Our results indicated that intermittent deep tillage practices helped to improve plant physiological growth status, chlorophyll a, and resistance to diseases, and the crop yield and value of output were increased with the deeper tillage practices. Crop yield (18.59%) and value of output (37.03%) were highest in IDT-50. There were three mechanisms of intermittent deep tillage practices that improved crop growth: (1) Intermittent deep tillage practices increased soil nutrients and root system architecture traits, which improved the fertility and nutrient uptake of crop through root system. (2) Changing rhizosphere environments, especially for root length, root tips, pH, and available potassium contributed to dissimilarity of bacterial communities and enriched plant growth-promoting species. (3) Functions associated with stress tolerance, including signal transduction and biosynthesis of other secondary metabolites were increased significantly in intermittent deep tillage treatments. Moreover, IDT-30 only increased soil characters and root system architecture traits compared with CK, but deeper tillage could also change rhizosphere bacterial communities and functional profiles. Plant height and stem girth in IDT-40 and IDT-50 were higher compared with IDT-30, and infection rates of black shank and black root rot in IDT-50 were even lower in IDT-40. The study provided a comprehensive explanation into the effects of intermittent deep tillage in plant production and suggested an optimal depth.

## Introduction

An increasing human population combined with decreasing resources, has created enormous pressure on agricultural producers to satisfy the increasing demand for food (Shah and Wu, 2019). To deal with the challenge, greater attention has been paid to increasing crop yields on the limited amount of arable land that is available. For many years, vast inputs of fertilizers, pesticides, and other chemicals were used on agriculture to improve agricultural production (Sharma and Singhvi, 2017). However, this has caused a series of severe environmental problems including soil health decline and land degradation. For example, excessive use of nitrogen fertilizer has clearly contributed to the emission of greenhouse gas, water eutrophication, and environmental degradation (Zhang et al., 2015). Therefore, efforts to ensure future higher crop productivity should be made with environmental sustainability in mind. Modern machinery and agricultural technologies have allowed farmers to make full use of limited land resources.

Smash-ridging tillage is a new farming method, which replaces traditional plowshare with a spiral drill (Wei, 2017). This machinery is able to cultivate a 30- to 50-cm depth of soil vertically through the use of high-speed peeling at 500–600 rounds per minute. The soil is both rotated and ground at the same time and then automatically deposited as a ridge. It could finally create greater amounts of loose soil, and the expansion of soil nutrients, moisture, oxygen, and microbial community (Zhang et al., 2020). Currently, deep vertical rotary tillage based on smash-ridging tillage has been applied to over 20 kinds of crops, including rice, wheat, tomato, sugarcane, and peanut, showing increased yields of 10–30% and quality improvement up to 5% (Wei, 2017). Research over the last decade has shown that improvement of soil structure increased the use ratio of crop to nutrients and promoted plant growth (Munkholm et al., 2013).

The assumption that promoting the availability of subsoil water and nutrition to plants, thereby improving crop yield through deep tillage, has a long history. The rapid development of steam and combustion engines allowed plowing depth to reach 200 cm in 1850 and 1960 (Eggelsmann, 1979). Since the 1970s, high costs and concerns about negative effects on soil structure and beneficial soil microbes increased resistance to the popularization and application of deep tillage in agriculture (Schneider et al., 2017). With respect to soil microbes, it is important to understand that deep tillage for ameliorative purposes can be performed annually to achieve gradual topsoil deepening over long-term shallow tillage. In some cases, ameliorative deep tillage has even showed positive impacts on plant growth by promoting rhizosphere microbes (Müller and Rauhe, 1959). However, the mechanisms between soil structure and crop health are still unclear, especially for the role of microbes in the process.

Rhizosphere bacteria are key mediators connecting plants health with the soil (Trivedi et al., 2020). Plants can establish complex and mutualistic relations with distinct microorganism by modulating the root environment. Several recent studies have shown that rhizosphere microbes displayed obvious preferences for rhizosphere soil as a source of metabolites and substrates. For example, banana root exudates, especially for organic acids, could help plant growth-promoting rhizobacteria (PGPR) strain *Bacillus amyloliquefaciens* NJN-6 colonize the host root (Yuan et al., 2015). A mass of microbes was attracted to root systems. Their metabolic activities and complex interactions with plants affect plant growth and productivity, via nutrient acquisitions, disease resistance, and stress tolerance (Philippot et al., 2013). However, these root and plant-associated microbes may induce host plant defense response by the plant immune system. Recently, it was revealed that root- and plant-associated bacteria contained a number of genes that mimic plant and root, and secrete effector proteins, in order to evade the plant immune system and successfully colonize the plant root (Nishad et al., 2020). Interactions between members of microbiome, such as cooperation and competition, also play an important role in the assembly process of the microbial community (Shi et al., 2016). Given the pivotal importance of rhizosphere environment–microbial interaction, assembling a beneficial rhizosphere microbial community via altered rhizosphere soil characteristics might be a potential way to increase crop growth.

Intermittent deep tillage (IDT, one-time deep tillage, and conventional tillage) through smash-ridging technology could promote agricultural production through optimizing soil quality without high disturbance. We hypothesized that changes in rhizosphere soil environment associated with different tillage practices would shape different bacterial community structures and changed bacterial community function profiles. To test our hypothesis, a field study was carried out to assess the improvement of IDT practices (30, 40, and 50 cm) on soil quality and crop growth. Soil properties, rhizosphere bacterial community, and plant performance in different tillage practices were analyzed to figure out the mechanism on how IDT practices promote crop growth through rhizosphere bacterial community and explore the effects of IDT depth.

## Materials and Methods

### Experimental Design

The experimental site is under conventional rotary tillage practices over 10 years and is located in Huayuan County, Hunan Province, China. The experiment had four treatments: intermittent deep tillage using a smash-ridging machine to a depth of 50 cm (IDT-50), intermittent deep tillage to a depth of 40 cm (IDT-40), intermittent deep tillage to a depth of 30 cm (IDT-30), and conventional rotary tillage with a depth of 20 cm (CK). Each treatment included three replicate fields. Before plant was transplanted, field soil was plowed and ridged according to the experimental design, with a width of 120 cm and a height of 30 cm. *Solanaceae* plants were then transplanted to a 100-m^2^ region at a planting density of 16,650 plants/hm^2^ on April 20, 2020. During the growth period, the agricultural management practices and fertilization regimes were similar for all plowing treatments.

### Sampling and Measurement

#### Rhizosphere Soil Physicochemical Characters

Mature period is the key period of *Solanaceae* quality formation, when soil bacterial communities play important roles. After 4 months, *Solanaceae* entered the mature stage of growth, and soil samples from plant rhizosphere soil were collected on August 2, 2020. We randomly selected two plants in each replicate field, and gently shook off the loosely adhered soil, then collected rhizosphere soil by brushing off soils with a sterile brush, which was tightly associated with the plant roots. Soil samples were divided into two parts for the following DNA extraction and physicochemical analysis, and stored at −80°C and 4°C, respectively. Soil pH, available phosphorus (AP), available potassium (AK), alkaline hydrolysable nitrogen (AN), and organic matter (OM) were measured according to the methods described by Zhang et al. (2019).

#### Plant Phytochrome Content in Leaves

Crop middle leaves (the tenth leaf) from the different treatments were collected to analyze plant phytochrome content in leaves. Fresh leaf samples were lyophilized and ground into powder to investigate the differences within leaf metabolites. Untargeted metabolome profiling was performed at the China Tobacco Gene Research Center using three platforms (Liu et al., 2019; Liu P. et al., 2020), including gas chromatography-mass spectrometry (GC-MS), liquid chromatography-mass spectrometry (LC-MS/MS), and capillary electrophoresis-mass spectrometry (CE-MS).

#### Crop Disease Infection Rate

Crop disease infection rates, including black shank, black root rot, bacterial wilt, and hollow stalk, were calculated by the percentage of infected plants in each field. That is,


Infectionrate=Ni/Nt×100%

Where Ni and Nt represent the number infected plants and the total number of plants in each field, respectively.

#### Crop Root System Architecture

The crop roots randomly chosen from different replicate fields were washed with sterilized water. Root system architecture (RSA) was obtained using an automatism scanner and image analyzer with WinRhizo REG system (Regents Instruments Inc., Ltd., Quebec City, Canada). The five RSA traits, including root length (RL), root projection area (RPA), root surface area (RSA), number of root tips (NRT), and number of root branches (NRB), were obtained broadly to describe the entire root system responses to different tillage practices (Luo et al., 2020).

#### Assessment of Crop Economic Value of Crop

The crop yield was determined by all the leaves of each treatment and then backed by local tobacco departments. Crop value of output was composed of the yield and average price (CNY, Chinese Yuan, generally referred to as RMB in China). The calculation formula of crop output value is as follows:


Cropvalueofoutput=cropyield×averageprice

Where Average price is obtained by dividing the total purchase value by the purchase weight after the local tobacco departments purchase the graded tobacco leaves (Formulated by State Tobacco Monopoly Administration of China).

### DNA Extraction and Illumina Sequencing

The total DNA of the rhizosphere soil was extracted using the FastDNA SPIN Kit for Soil (MP Biomedicals, Santa Ana, CA, United States). Extracted DNA quality was assessed by NanoDrop ND-2000 spectrophotometer (ND-1000 Spectrophotometer, United States) according to 260/280- and 260/230-nm absorbance ratios. Amplification of the V3–V4 hypervariable region of the bacterial 16S rRNA gene was performed with the primers 341F (5′-CCTACGGGNGGCWGCAG-3′) and 805R (5′-GACTACHVGGGTATCTAATCC-3′). The PCR amplifications were combined and sequenced on Illumina MiSeq at LC Sciences (Hangzhou, China). The 16S rRNA gene sequences have been submitted to the NCBI SRA database, and the project number is PRJNA692565.

The sequencing data were processed on an in-house galaxy pipeline^1^ developed in the lab of Dr. Zhou (Institute for Environmental Genomics, University of Oklahoma). First, bases after ambiguous bases (N) were removed from the sequences, and then the sequences were trimmed based on the length. After removing chimeras and singletons, reads with 97% similarity were then assigned to the same out, and an OTU table was generated using UPARSE (Edgar, 2013). Taxonomic assignment was performed by blasting the sequences against the Ribosomal Database Project (RDP) database with a 50% minimal confidence (Wang et al., 2007). Finally, a rarefied OTU table was created at a depth of 10,000 reads per sample to eliminate the influence caused by different sequencing depths. All downstream analyses were carried out using the normalized OTU table.

### Data Analyses

The phylogenetic community dissimilarity base on UniFrac distance among treatments was calculated and plot using “*phyloseq*” and “*ggplot2*” in R (version 3.6.3) (McMurdie and Holmes, 2013). Principal coordinate analysis (PCoA) was used to ordinate the samples. The community dissimilarity analyses (β-diversity) based on Bray–Curtis and Euclidean distance, the α-diversity of community, including Chao value, Shannon index, Simpson index, species richness and Pielou’s evenness, and Mantel test based on Spearman’s correlation of environmental variables with bacterial community structure were performed using the “*vegan*” package in R. Then compositional dissimilarities among treatments (β-diversity) were partitioned into replacement and richness difference components through the Jaccard dissimilarity index using the R package *adespatial* (Legendre and De Caceres, 2013). The contributions of rhizosphere soil physicochemical characteristics and root structure parameters to bacterial community variation were also done by variance partitioning canonical correspondence analysis (VPA) via *vegan* R package. The OTUs with relative abundances significantly different between the rhizosphere soils of deep tillage treatments and conventional tillage treatment were detected by likelihood ratio test (LRT) through R package *DESeq2* (Love et al., 2014) and adjusted *p*-value using the false discovery rate (FDR) method. Venn diagram and heatmap analyses were conducted using the “*venndiagram*” and “*pheatmap*” package in R. A one-way ANOVA followed by Tukey’s test was used to calculate the significance of differences among treatments. All statistical analyses were performed using the R software.

Molecular ecological network based on OTU table, rhizosphere soil physicochemical characteristics, and root structure parameters was constructed to represent their co-occurrence pattern (Zhou et al., 2011; Meng et al., 2019). The network analyses were performed using an online pipeline^2^ according to a previous study. Only OTUs present in at least 12 out of 24 samples were used for network construction. The random matrix theory (RMT) was used to choose the similarity threshold (*St*) automatically before network construction. The network was visualized using Cytoscape software (version 3.8.1). To assess possible topological roles of taxa in the network, the nodes were classified into four different categories based on their within-module connectivity (Z_*i*_) value and among-module connectivity (P_*i*_) value: module hubs (Z_*i*_ ≥ 2.5), network hubs (Z_*i*_ ≥ 2.5 and Pi ≥ 0.62), connectors (Pi ≥ 0.62), and peripherals (Zi < 2.5 and Pi < 0.62). To define the distribution of OTUs in four treatments, we classified all OTUs into two categories: shared OTUs in four treatments (AET) and special OTUs only existing in deep tillage treatments (AT).

The Phylogenetic Investigation of Communities by Reconstruction of Unobserved States (PICRUSt2)^3^ was applied to predict potential functional profiles of the bacterial community using 16S rRNA gene data (Douglas et al., 2020). First, various gene contents in each OTU in the reference phylogenetic tree were calculated, and the abundance table of the predicated gene family can be established. Then, the resulting gene content predictions of all OTUs with the relative abundance table of OTUs in the microbial community were combined, and the predicated abundances of gene families in the community were generated. Finally, the KEGG database was used to predicate functional information at different pathway levels. The Bray–Curtis distance-based PCA revealing the functional structure among treatments based on the KO table was constructed using the “vegan” package in R. The comparison of function gene at levels 1 and 3 was conducted using *t*-test in STAMP.

Random forest analysis to identify the major statistically significant microbial predictors was conducted with the “*randomForest*” package (Liaw and Wiener, 2002; Chen et al., 2020). A total of 35 OTUs, important in assigning samples, were selected in random forest modeling. The importance of each OTU in distinguishing different treatments was assessed by calculating the average decline in module accuracy when the OTU was removed from the community.

## Results

### Yield and Statistics of Crops in Different Tillage Treatments

After deep tillage practices, the crop yield and output value were both increased (Table 1). The crop values of output were 58,609.52, 54,306.25, 48,887.20 and 42,770.56 CNY/hm^2^ in IDT-50, IDT-40, IDT-30, and CK, respectively. The yields of crop were 2,410.71, 2,294.64, 2,142.86, and 1,897.22 kg/hm^2^ in IDT-50, IDT-40, IDT-30, and CK, respectively. The average prices were 24.31, 23.67, 22.81, and 22.54 CNY in IDT-50, IDT-40, IDT-30, and CK, respectively. Thus, the crop yield and output value in IDT-50 were, respectively, 27.07 and 37.03% higher than those in CK. In IDT-40, crop yield and output value were, respectively, 20.95 and 26.97% higher than those in CK. In IDT-30, crop yield and output value were, respectively, 12.95 and 14.30% higher than those in CK.

**TABLE 1:** Yield, average price, and output value of crop among treatments.

Sample	Average price (CNY/kg)	Crop yield (kg/hm^2^)	Crop value of output (CNY/hm^2^)
IDT-50	24.31	2410.71	58609.52
IDT-40	23.67	2294.64	54306.25
IDT-30	22.81	2142.86	48887.20
CK	22.54	1897.22	42770.56

### Effects of Intermittent Deep Tillage on Crop Rhizosphere Environment

**FIGURE 1:**
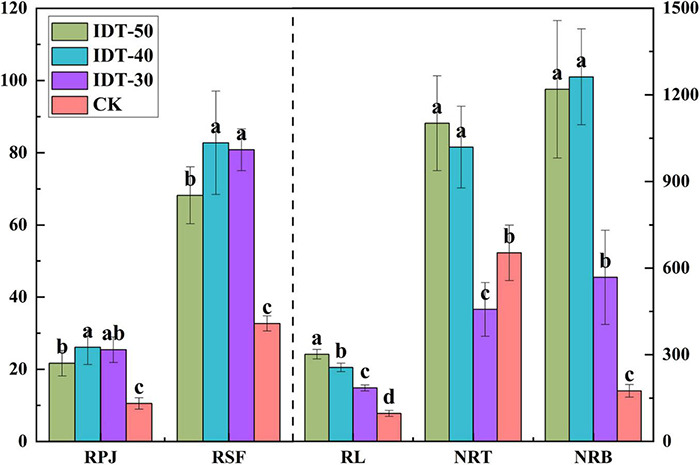
One-way ANOVA of root system architecture (RSA) traits among treatments. Different letters indicate statistically significant differences among treatments (*p* < 0.05). RPJ, root projection area (cm^2^); RSF, root surface area (cm^2^); RL, root length (cm); NRT, number of root tip; NRB, number of root branches.

Rhizosphere soil physicochemical were significantly different among different treatments. Significant decreases in pH and AN, and increase in AP, AK, and OM, were observed in IDT treatments compared with CK (Tukey’s test, *p* < 0.05) (Table 2). Besides, different depths of IDT caused different effects on rhizosphere soil. IDT-40 treatment caused largest increase in AK and OM, and decrease in pH and AN. AP was highest in IDT-50 and IDT-30. Root growth characteristics were also analyzed and found that RPA, RSF, RL, NRT, and NRB were all significantly increased (Tukey’s test, *p* < 0.05) in IDT treatments compared with CK, except for NRT, which showed a significant decrease in IDT-30 (Tukey’s test, *p* < 0.05) (Figure 1). RL increased regularly and significantly (Tukey’s test, *p* < 0.05) with IDT depth (IDT-50 > IDT-40 > IDT-30); NRT and NRB were more in IDT-50 and IDT-40 than IDT-30; RSA was larger in IDT-40 and IDT-30; RPJ was largest in IDT-40. In summary, RSA traits were increased with the increasing IDT depth, especially for RL, but NRB and NRT were increased the most when IDT depth reached 40 cm, and RPJ and RSF were even decreased when depth was over 40 cm.

**TABLE 2:** Plant rhizosphere soil physicochemical analysis among treatments.

Sample	pH	AP (mg kg^–1^)	AK (mg kg^–1^)	AN (mg kg^–1^)	OM (mg kg^–1^)
IDT-50	6.067 ± 0.027b	26.022 ± 0.280a	137.79 ± 1.42b	165.63 ± 3.24b	20.063 ± 0.234b
IDT-40	5.773 ± 0.019d	23.889 ± 0.376b	143.29 ± 1.88a	121.14 ± 5.00d	26.935 ± 0.180a
IDT-30	5.950 ± 0.028c	26.467 ± 0.317a	142.96 ± 1.87a	148.67 ± 5.83c	19.454 ± 0.158c
CK	6.115 ± 0.023a	17.261 ± 0.476c	130.18 ± 3.48c	239.63 ± 5.08a	18.562 ± 0.048d

*AP, available phosphorus; AK, available potassium; AN, alkali-hydrolyzed nitrogen; OM, organic matter.*

*Values are mean ± standard error of six replicate soil samples per treatment.*

*Different letters indicate statistically significant differences within rows (one-way ANOVA Turkey’s test, p < 0.05).*

### Rhizosphere Bacterial Community Diversity

A total of 1,747,753 high-quality sequences were obtained from high-throughput sequencing and clustered into 3,822, 3,796, 3,518, and 3,768 OTUs in IDT-50, IDT-40, IDT-30, and CK, respectively. The alpha diversity of rhizosphere bacterial community, including Chao value, Simpson index, Shannon index, species richness, and Pielou’s evenness, had no significant difference in IDT treatments compared with CK (Supplementary Table 1), but the increasing depth of IDT led to significant increases in Chao value and species richness among three level IDT treatments (Tukey’s test, *p* < 0.05). The PCoA results showed that there were significant differences in beta diversity of rhizosphere bacterial community based on the Bray–Curtis dissimilarity matrix (Supplementary Figure 1A, ANOSIM = 0.524, *p* = 0.001), unweighted UniFrac distance (Supplementary Figure 1B, ANOSIM = 0.521, *p* = 0.001), and weighted UniFrac distance (Supplementary Figure 1C, ANOSIM = 0.326, *p* = 0.001). For further analysis of differences in beta diversity, significance tests were performed, including MRPP and ANOSIM calculated with Euclidean or Bray–Curtis distance (Table 3). The results showed that the beta diversity of IDT-50 and IDT-40 were significantly different from IDT-40 and CK, but that there was no significant difference between IDT-50 and IDT-40, and between IDT-30 and CK based on Euclidean distance. Additionally, the beta diversity decomposition analyses showed that the bacterial community compositional dissimilarities among four treatments were dominated by species replacement processes (contributed 88.46%), while richness difference processes only contributed 11.54% (Supplementary Figure 2).

**TABLE 3:** Significance tests on the effects of different tillage methods on the rhizosphere bacterial community structure.

Sample	Distance	CK		IDT-50		IDT-40	
		ANOSIM	MRPP	ANOSIM	MRPP	ANOSIM	MRPP
IDT-50	Bray–Curtis	0.907**	0.341**				
	Euclidean	0.367**	4.041**				
IDT-40	Bray–Curtis	0.637**	0.353**	0.407**	0.331**		
	Euclidean	0.235**	4.589**	0.054	4.205		
IDT-30	Bray–Curtis	0.430*	0.389*	0.648**	0.367**	0.422**	0.379**
	Euclidean	0.131	5.868	0.391**	5.484**	0.215*	6.032*

*Two different permutation tests were performed, including the multiple response permutation procedure (MRPP) and analysis of similarity (ANOSIM) calculated with Euclidean or Bray–Curtis distance.*

*Bold values indicate significant difference.*

*p < 0.05 and **p < 0.01.

**FIGURE 2:**
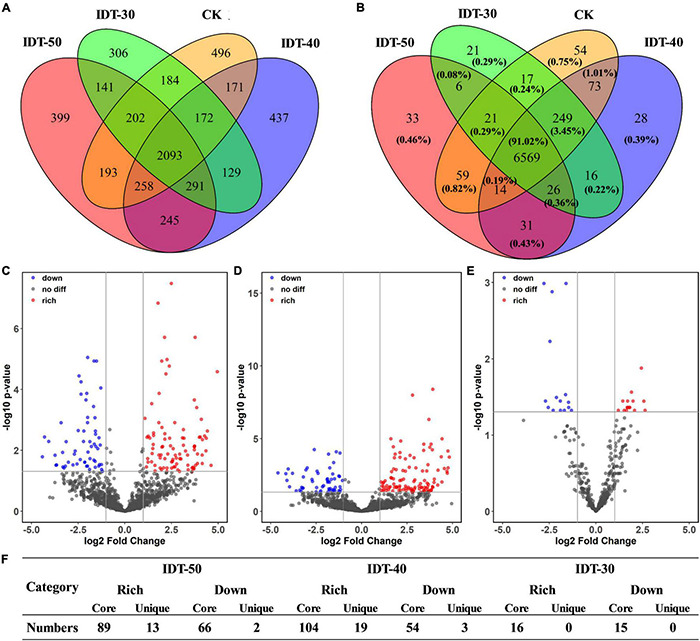
The effects of different tillage methods on rhizosphere microbial compositions. The overlap of taxonomical OTUs **(A)** and functional KOs **(B)** in conventional tillage treatment and intermittent deep tillage (IDT) treatments. Volcano plots **(C–E)** represent the community compositions in IDT-50, IDT-40, and IDT-30, respectively. The rhizosphere bacterial OTUs with relative abundances significantly (log2 fold change > | 1.5| and FDR adjusted *p*-value < 0.05) different between IDT (red) and CK treatments are colored in the volcano plot. **(F)** Statistic analysis for the OTUs with relative abundances significantly different between IDT and CK treatments.

Likelihood ratio test results revealed that rhizosphere soil in different tillage treatments harbored distinct bacterial community compositions with unique respective sets of OTUs. Venn diagram revealed that the number of core bacterial OTUs was 2,093, which accounted for 93.02, 93.21, 94.25, and 93.74% of the rhizosphere soil sequences in IDT-50, IDT-40, IDT-30, and CK, respectively (Figure 2A). The remaining OTUs included unique species and overlap species, which were all rare species with relative abundance lower than 0.1%. LRT results showed that the relative abundance of 102 OTUs, 123 OTUs, and 16 OTUs were richer compared with CK in IDT-50, IDT-40, and IDT-30, respectively (*p*_*adj*_ < 0.05), and 68 OTUs, 57 OTUs, and 15 OTUs were lower compared with CK in IDT-50, IDT-40, and IDT-30, respectively (*p*_*adj*_ < 0.05) (Figures 2C–E). Furthermore, there were only 13, 19, and 0 unique OTUs (only detected in IDT treatments) enriched significantly in IDT-50, IDT-40, and IDT-30, respectively (Figure 2F). In other words, more than 90% of the OTUs, whose relative abundance changed significantly compared with CK, were core species in four treatments. In summary, IDT caused the species replacement of rare species and richness difference of core species in rhizosphere bacterial communities, and there were most changed OTUs at relative abundance in IDT-40.

### Rhizosphere Environment Affected the Reassembled Bacterial Community

**FIGURE 3:**
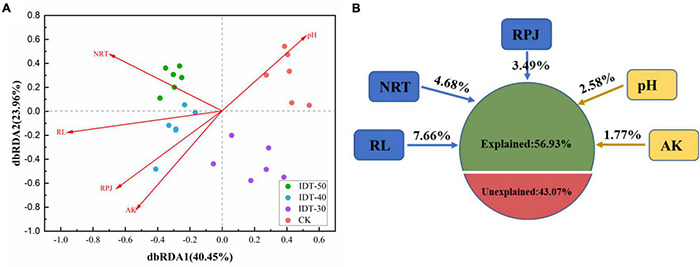
Distance-based (distance = Bray) redundancy analysis (dbRDA) **(A)** depicting the correlation between bacterial communities and rhizosphere environment, and **(B)** variance decomposition, with the percentages of variance explained by s soil physicochemical and plant root growth characteristics. AK, available potassium; RPJ, root projection area (cm^2^); RL, root length (cm); NRT, number of root tip.

Spearman’s correlation analysis by the Mantel test found that the rhizosphere environment, including both soil physicochemical and crop root growth characteristics, had significant effects on rhizosphere bacterial community (Table 4). Furthermore, the distance-based redundancy analysis (db-RDA) was performed to assess the extent to which the rhizosphere environmental parameters explained the variance of bacterial community in the four tillage treatments. Figure 3 showed that all of these factors together explained 56.93% of community variances. After selecting the most parsimonious explanatory variables, five environmental factors including RL, NRT, RPA, pH, and AK were retained. Across all treatments, RL was the most remarkable, followed by NRT, RPA, pH, and AK.

**TABLE 4:** Spearman’s correlation of environmental variables with bacterial community structure as determined by the Mantel test.

Variable	*r*	*p*
RL	**0.5473**	0.001
RPJ	**0.3029**	0.004
RSF	**0.3938**	0.001
NRT	**0.3456**	0.001
NRB	**0.4919**	0.001
pH	**0.1595**	0.033
AP	**0.3505**	0.002
AK	**0.3260**	0.002
AN	**0.3881**	0.001
OM	**0.2434**	0.006

*Bold values indicate significant difference.*

*AP, available phosphorus; AK, available potassium; AN, alkali-hydrolyzed nitrogen;*

*OM, organic matter. RPJ, root projection area (cm^2^); RSF, root surface area (cm^2^);*

*RL, root length (cm); NRT, number of root tip; NRB, number of root branches.*

**FIGURE 4:**
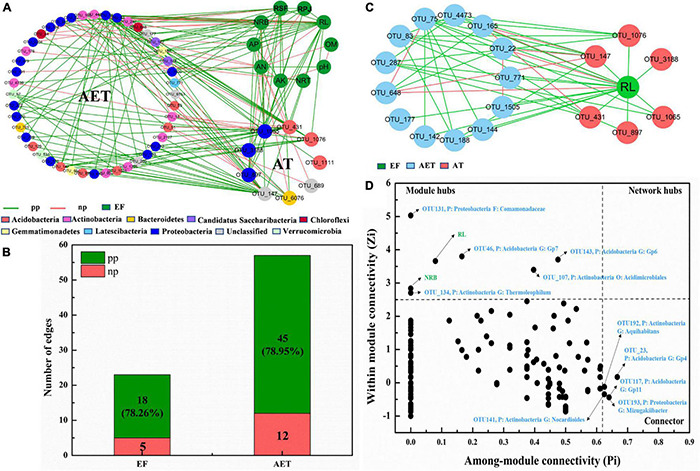
Random matrix theory (RMT)-based molecular ecology networks (MENs). **(A)** Nodes (OTUs) connected to “AT” and “EF.” Green lines represent the interactions between nodes (OTUs) that are negative, and red lines represent positive interactions. EF, rhizosphere environmental variations. **(B)** Numbers of edges for AT connecting to EF and AET. AET, OTUs shared in four treatments; AT, special OTUs present in deep tillage treatments. pp, positive Pearson correlation; np, negative Pearson correlation. **(C)** Nodes (OTUs) connected to “RL”. **(D)** *Zi–Pi* graph showing “keystone” OTUs. P, phylum; G, genus.

To investigate how rhizosphere environmental factors affected bacterial community composition, an overall network based on OTU abundance, soil physicochemical, and plant root growth characteristics was constructed (Supplementary Figure 3A). Topological properties of the network are presented in Supplementary Table 2. There were 42 modules including 347 nodes and 647 edges (Supplementary Table 2). All environmental factors, except NRT, were grouped in the same module, module 2, and a subnetwork was constructed with OTUs connected to “AT” and “EF” (Figure 4A). Furthermore, as seen in Figure 4B, AT had 78.26 and 78.95% positive edges with EF and AET in the whole network. The most important environmental factor, RL, showed positive correlation with AT and most AET, except for OTU_648 (*Sphaerobacter*), OTU_22 (*Bradyrhizobium*), and OTU_165 (*Conexibacter*) (Figure 4C). Additionally, the three OTUs (OUT_648, OUT_22, and OUT_165) showed negative correlations with OTU_147 (*Unclassified*) and OTU_431 (*Gp6*) in the AT group. Supplementary Figure 3B also showed that NRB only had negative relationships with OTU_22 and OTU_648 in AET, which showed a negative correlation with OTU_147 in AT. The *Zi–Pi* graph showed the roles of OTUs played by the OTUs in the community (Figure 4D). There were seven “module hubs” and five “connectors.” Two rhizosphere environmental factors (RL and NRB) were module hubs and played important roles in the rhizosphere bacterial community.

### Relationship Between Bacterial Community and Crop Growth Traits

Crop growth traits including disease infected rate, plant height, stem girth, and the content of leaf pigment were investigated to evaluate the effects of IDT to crop growth (Table 5). Based on ANOVA, crop physiological growth status including plant height and stem girth in IDT-50 and IDT-40 were significantly higher than in CK, and plant height in IDT-30 was also higher than in CK (*p* < 0.05). Contents of chlorophyll a in IDT treatments were also significantly higher than in CK (*p* < 0.05). Disease infection rate of black root rot, bacterial wilt, and hollow stalk in IDT treatments showed significant decreases compared with those in CK (*p* < 0.05). Further analysis showed that the control efficiency of IDT to bacterial wilt was best, and reached 88.29, 77.42, and 70.29% in IDT-50, IDT-40, and IDT-30, respectively, and the worst control efficiencies to Hollow stalk were also reached up to 68.86, 68.81, and 50.02% in IDT-50, IDT-40, and IDT-30, respectively. In other words, the plant growth-promoting effect in IDT-50 was best among three level IDT practices, but IDT-40 also showed better performance in enhancing plant height and stem girth.

**TABLE 5:** Plant performance including plant physiological growth status, disease infection rate, and pigment content respond to different tillage treatments.

	Trait	IDT-50	IDT-40	IDT-30	CK
Plant physiological	**Plant height**	**120.18 ± 3.70a**	**116.67 ± 4.29a**	**110.53 ± 2.09b**	**105.53 ± 1.17c**
	**Stem girth**	**9.87 ± 0.33a**	**9.90 ± 0.21a**	**9.30 ± 0.14b**	**9.43 ± 0.16b**
Disease infection rate (%)	**Black shank**	**1.05 ± 0.33c**	**2.03 ± 0.46bc**	**2.67 ± 0.76b**	**8.99 ± 1.50a**
	**Black root rot**	**0.57 ± 0.51c**	**1.70 ± 0.80bc**	**2.84 ± 1.30b**	**8.51 ± 1.34a**
	**Bacterial wilt**	**0.57 ± 0.62b**	**0.57 ± 0.51b**	**1.15 ± 0.37b**	**9.18 ± 2.15a**
	**Hollow stalk**	**1.07 ± 0.34b**	**1.07 ± 0.48b**	**1.72 ± 0.51b**	**3.44 ± 0.63a**
Pigment content	Neoxanthin	72.74 ± 11.04a	66.85 ± 7.27a	64.52 ± 7.18a	73.14 ± 10.26a
	Violaxanthin	33.70 ± 9.74a	49.05 ± 10.14a	40.19 ± 11.63a	34.31 ± 7.56a
	Lutein	987.10 ± 116.00a	952.70 ± 115.20a	963.20 ± 127.00a	906.80 ± 125.30a
	Chlorophyll b	478.36 ± 142.89a	444.33 ± 81.91a	430.10 ± 51.80a	461.69 ± 69.71a
	**Chlorophyll a**	**1357.60 ± 485.30a**	**1419.00 ± 261.50a**	**1302.90 ± 144.50a**	**558.5 ± 106.00b**
	Carotenoid	130.97 ± 19.80a	125.18 ± 12.79a	125.21 ± 10.11a	128.20 ± 10.59a

*Values are presented as mean ± SD (n = 6), bold values with different letters indicate significant difference by one-way ANOVAs (Tukey’s test at p < 0.05).*

**FIGURE 5:**
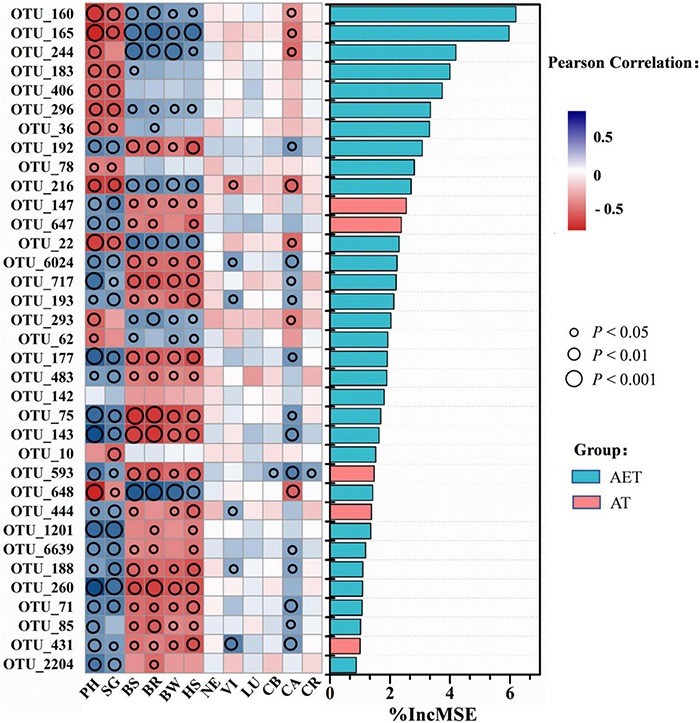
Correlation and best random forest model for the relative abundance of top 35 most important taxa. For variable selection and estimating predictability, the random forest machine-learning algorithm was used. Circle size represents the *p*-value as a result of Pearson correlation analysis between taxa and plant growth traits including plant physiological growth status, disease morbidity, and pigment contents. Different colors represent Pearson correlations. Plant physiological growth status: PH, plant height; SG, stem girth. Disease morbidity: BS, black shank; BR, black root rot; BW, bacterial wilt; HS, hollow stalk. Leaf pigment contents: NE, neoxanthin; VI, violaxanthin; LU, lutein; CB, chlorophyll b; CA, chlorophyll a; CR, carotenoid.

Through random forest analysis, the top 35 OTUs in distinguishing between different tillage treatments were obtained by modeling. Pearson correlation showed that these OTUs influenced plant growth traits significantly (*p* < 0.05), expected for OTU_142 (Figure 5). Additionally, 20 OTUs showed positive effects on plant growth, which were positively related to plant characteristics including plant height, stem girth, leaf pigment, or negatively related to plant disease incidence. These OTUs are mainly distributed in the genera *Aeromicrobium*, *Aquabacterium*, *Aquihabitans*, *Gemmatimonas*, *Hydrogenophaga*, *Gp3*, *Gp6*, *Kofleria*, *Mizugakiibacter*, *Saccharibacteria_genera_incertae_sedis*, and *Streptomyces* (Supplementary Table 3). The 14 OTUs showing negative effects on plant growth were mainly classified into *Thermoleophilum*, *Conexibacter*, *Gaiella*, *Sphaerobacter*, and *Bradyrhizobium*. After LDA analysis, the 20 OTUs (expected for OTU_1201) that showed positive effects on plant growth traits in IDT treatments were enriched compared with CK at relative abundance, while other OTUs showing negative effects were decreased on the contrary (Supplementary Table 3). In addition, five OTUs, including OTU_147 (Unclassified), OTU_647 (*Saccharibacteria_genera_incertae_sedis*), OTU_593 (*Gp3*), OTU_444 (Unclassified), and OTU_431 (*Gp6*), were only detected in deep tillage treatments.

### Reassembled Rhizosphere Bacterial Communities Were Functionally Divergent

In order to investigate the rhizosphere functions, metagenomes of the bacterial communities were predicted using PICRUSt and then annotated by referring to KEGG databases. A total of 7,218 KEGG orthologs were assessed in the four treatments. PCoA analysis (Supplementary Figure 4A) at the KEGG ortholog level showed that there were obvious differences among function terms in the four treatments (ANOSIM R = 0.176, *p* = 0.005). Furthermore, ANOSIM and MRPP based on Bray–Curtis distance showed that the function terms of IDT-50 and IDT-40 differed significantly from those of IDT-30 and CK, whereas there was no obvious difference between those of IDT-50 and IDT-40 or between those of IDT-30 and CK (Table 6). A Venn diagram (Figure 2B) showed that IDT did not change the overall functions found in the rhizosphere soil bacterial community, but different level IDT treatments were enriched in particular functions.

**TABLE 6:** Significance tests on the effects of different tillage methods on the rhizosphere bacterial functional profiles.

Sample	Distance	CK		IDT-50		IDT-40	
		ANOSIM	MRPP	ANOSIM	MRPP	ANOSIM	MRPP
IDT-50	Bray–Curtis	0.259*	0.036*				
IDT-40	Bray–Curtis	0.193*	0.039*	−0.024	0.03		
IDT-30	Bray–Curtis	0.052	0.05	0.470**	0.040**	0.309*	0.044**

*Two different permutation tests were performed, including the multiple response permutation procedure (MRPP) and analysis of similarity (ANOSIM) calculated with Bray–Curtis distance.*

*Bold values indicate significant difference.*

*p < 0.05 and **p < 0.01.

**FIGURE 6:**
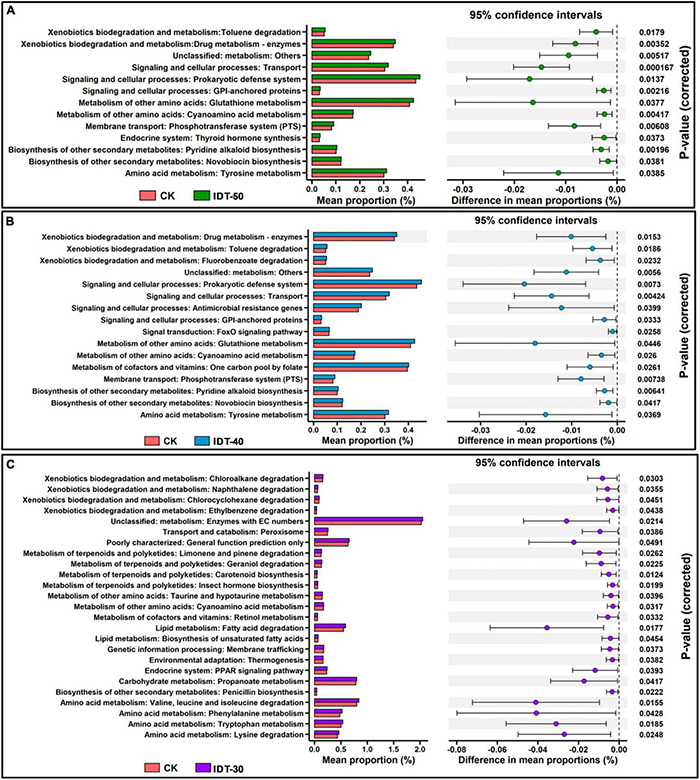
PICRUSt predicted metagenome functions at level 3. **(A)** *Post hoc* plot showing KEGG level 3 function terms that differed significantly between IDT-50 and CK. **(B)** *Post hoc* plot showing KEGG level 3 function terms that differed significantly between IDT-40 and CK. **(C)** *Post hoc* plot showing KEGG level 3 function terms that differed significantly between IDT-30 and CK.

After comparisons with the KEGG database, eight categories of biological metabolic pathways (level 1) were obtained (Supplementary Figure 4). Among them, rare terms “Organismal Systems” and “Human Diseases” were significantly higher in IDT-50 and IDT-40 than in CK (*t*-test, *p* < 0.05), the abundant term “Environmental Information Processing” was higher in IDT-50, and “BRITE Hierarchies” was higher in IDT-40. However, there was no significant difference of function term at level 1 between IDT-30 and CK (*t*-test, *p* < 0.05). For further analysis, 453 categories of biological metabolic pathways (level 3) were obtained, and abundant functions (number of genes >100) in IDT treatments were selected and compared with the CK based on *t*-test (Figure 6). There were 25, 16, and 13 categories of biological metabolic pathways enriched in IDT-30, IDT-40, and IDT-50, respectively. In IDT-50 and IDT-40, functions about xenobiotics biodegradation and metabolism, signaling and cellular processes, metabolism of amino acid, biosynthesis of other secondary metabolites, and membrane transport were enriched. In IDT-30, the enriched functions were mainly related to xenobiotic biodegradation and metabolism, terpenoid and polyketide metabolism, lipid degradation, and amino acid metabolism. Besides, 12 enriched categories in IDT-50 were also enriched in IDT-40, and the differences in mean proportions with 95% confidence intervals were similar. For example, the difference of prokaryotic defense system (signaling and cellular processes at level 2) in proportions was 0.017% between IDT-50 and CK, and 0.020% between IDT-40 and CK; the difference of transport (signaling and cellular processes at level 2) in proportions was 0.015% between IDT-50 and CK, and 0.014% between IDT-40 and CK, but only one category (metabolism of other amino acids: cyanoamino acid metabolism) in IDT-50 and IDT-40 was also enriched in IDT-30.

## Discussion

Long-time conventional rotary tillage in shallow layer have generated increasingly serious soil degradation problem to agriculture industry (Hou et al., 2012), which increased soil bulk density in deep soil layer and decreased plant availability to soil fertility and water (Hu et al., 2021). Intermittent deep vertical rotary tillage (IDT) was tried creatively to address this issue in Chinese agriculture (Schneider et al., 2017). In this study, soil fertility and crop growth traits were all improved in three-level IDT treatments compared with those in CK. Besides, the crop yield and output value were increased with the increasing of IDT depth. The results confirmed our hypothesis and indicated that crop growth traits were closely related to IDT depth. When IDT depth reached 40 cm, crops would be stronger with the highest plant height and thickest stem girth. Deeper IDT practice to 50 cm would not bring better improvement on crop physiological growth status, but there is reduced disease morbidity. With responses of soil characters, rhizosphere bacterial community, and crop traits in the mature period of crops, the mechanism by which intermittent deep tillage promoted plant growth was tried to be revealed.

### Intermittent Deep Tillage Increased Soil Fertility and Nutrition Acquisition Through Roots

Tillage practice in agriculture would affect field soil aeration and fertility directly, which was closely related to crop growth. Long-term shallow tillage with conventional rotary tillage to a 20-cm soil depth causes the plow layer to narrow, hindering the flow of air and water (Liu Z. et al., 2020). IDT practices alter soil structure including soil-specific surface area and increasing soil porosity to be more suitable for crop root growth (Helliwell et al., 2019). Moreover, our study found that RSA traits, which reflect crop nutrient uptake and competition ability, were increased with the increase in IDT depth and highest in IDT-40 (Figure 1). Previous deep tillage research to 50 cm had found that root DMA in 0–40 cm of soil layer significantly increased, but the effect was not obvious in the deep soil layer (Zhai et al., 2017). Thus, IDT-50 could further increase root length, but RSF, RPJ, NRT, and NRB would not increase. These phenomena were also found in aboveground crop growth traits, such as plant height and stem girth (Table 5). Meanwhile, soil fertility properties in crop rhizosphere soil were significantly increased significantly in rhizosphere soil under IDT treatments. Potassium and phosphorus are essential macronutrients for crop growth and development, which help to enhance the regulation of host crop to the environment and improve the resistance of crops to cold, drought, and diseases (Prajapati and Modi, 2012; Malhotra et al., 2018). Thus, a decrease in disease morbidity and increase in bacterial functional gene stress tolerance in deep tillage treatments might be closely related with increase in AP and AK in soil. Potassium is also beneficial for plant to take up nitrogen (Divito and Sadras, 2014), which is an important component of chlorophyll (Piekkielek and Fox, 1992). Therefore, the increase in AK in soil indirectly promotes the synthesis of chlorophyll in crops. However, rhizosphere soil physicochemical characters were not related regularly with IDT depth. For example, AK was highest in IDT-40 and IDT-30, but AP was highest in IDT-40 and IDT-50. Overall, IDT practices could help to improve soil fertility and nutrient uptake ability through the roots, and deeper IDT would further increase the RSA traits.

### Intermittent Deep Tillage Affected Bacterial Community Assembly Through Rhizosphere Environment

Tillage management is an indispensable practice in agricultural production, but one can also induce alterations in the soil microbial community (Govaerts et al., 2008). Microbes play an important role in the soil ecosystem and are a bioindicator of cropland soil quality (Onet et al., 2019). In the present study, bacterial community diversity of the plant rhizosphere was significantly altered with the different tillage methods, but the responses were quite different. IDT treatment did not change bacterial community diversity compared with conventional tillage, but increasing IDT depths increased species richness. A previous study found that vertical profiles of microbial communities were present in cropland soil (Eilers et al., 2012), which meant different community compositions caused by different soil depths. Tillage management allows microbes from deeper soil layers, different from the microbes present in the conventional tillage layer, to colonize the upper layer through the turning and mixing of soil horizons (Schneider et al., 2017). Meanwhile, the environment of the plant rhizosphere presents a selective pressure on the microbial community (Trivedi et al., 2020), with niche filtering having a great effect on the survival of bacteria (Shi et al., 2016). Thus, most species from deeper soil layers were filtered with only few species able to survive in the crop rhizosphere. Thus, differences in community beta-diversity were observed not only between different tillage methods but also between different IDT treatments in our study. Microbial community beta-diversity can be decomposed into the process of species replacement and species richness differences (Legendre and De Caceres, 2013). Our results showed that crop rhizosphere community compositional dissimilarities were dominated by species replacement and became less divergent when the relative abundance of species was considered. This meant that the presence/absence of bacterial species played a more important role in bacterial community variation than changes in the relative abundance. Several studies have found that niche difference and interspecific competition determine the presence/absence and relative abundance of bacterial species and affect the bacterial community (Liao et al., 2016; Li et al., 2019). In summary, IDT introduced some species into surface layer soil that then survived in the new environment after fierce competition with resident species, causing significant dissimilarity in the bacterial community. Therefore, there is a great need to increase our understanding on how different tillage practices affect interactions between bacterial species and drive the assembly of the rhizosphere bacterial community.

Plants recruit microbes from soil and endophytic communities to the rhizosphere to establish complex and mutualistic relations with distinct microorganism through modulation of the root environment (Yu L. et al., 2019; de Vries et al., 2020; Ren et al., 2020; Wan et al., 2020). In this study, rhizosphere environmental factors, especially RL, NRT, RPJ, and pH, affected the assembly process of the rhizosphere bacterial community in our study. Consistent with our result, studies in forests and paddy fields (Chen et al., 2019; Yu K. et al., 2019; Merino-Martín et al., 2020) have found that rhizosphere bacterial communities are strongly related to plant root structure. A stronger root system could transport more oxygen, water, and nutrients into the soil, improve soil aeration, and maintain high root oxidation, which contributes to more suitable conditions for microbial activity and growth (Merino-Martín et al., 2020). Furthermore, crop roots form a large and complex network with microbes, which could help explain how various rhizosphere environmental factors affect interactions between bacterial species and drive the functional shifts within the bacterial community. RSA traits, including RL and NRB, work as a strong environmental filter during rhizosphere bacterial community assembly (Shi et al., 2016; Saleem et al., 2018; Merino-Martín et al., 2020). Then root exudates would be changed accompanied with root system architecture (Saleem et al., 2018), and root exudates may aid the growth and colonization of species from deep soil layers within the plant rhizosphere. Recent studies have shown that root exudates, such as arabinogalactan proteins (AGPs), benzoxazinoids, peroxidases, and glycosyl hydrolase family 17, are able to attract potentially beneficial microbes and repel plant root pathogens (Cannesan et al., 2012; Neal et al., 2012; Huang et al., 2014). They also help to explain why the enriched beneficial taxa are located in the key positions of the network and strongly affected the other nodes (Jiao et al., 2019). Overall, network analysis provided information for us to postulate that rhizosphere environmental factors, especially for crop root traits, exert great influence on the network structure of the rhizosphere bacterial community, which ultimately caused changes in community composition.

### Intermittent Deep Tillage Promoted Crop Growth Through Rhizosphere Soil Bacterial Community

Deep tillage practice changed rhizosphere bacterial community through species replacement processes of rare species and richness difference processes of core species (Shen et al., 2020), which eventually revealed the mechanism of plant growth promotion through rhizosphere bacteria. We detected that replacing species that were detected in IDT treatments significantly affected crop growth, including plant height, stem girth, disease morbidity, and contents of leaf pigment. In addition, core species that showed positive effects on crop growth were enriched in IDT treatments, while species that showed negative effects on crop growth were decreased. For example, enriched species, such as OUT_143, OTU_85, OTU_483, and OTU_188, were classified into *Gp6*, *Aeromicrobium*, *Aquabacterium*, and *Gemmatimonas*. Studies found that *Aeromicrobium* increased tolerance of host plant to abiotic stress, and improved growth, yield, and biomass (Martin and Dombrowski, 2015); *Aquabacterium* and *Gemmatimonas* were found to be phosphate-solubilizing bacteria in Taro and maize rhizosphere (Yang et al., 2017; Aloo et al., 2020); *Acidobacteria Gp6* was usually present in higher abundance in the nutrient-enriched soil (Sul et al., 2013). Replacing species OTU_431 classified into *Gp6* also promoted plant growth and decreased disease morbidity. However, decreased species OTU_160, OTU_296, and OTU_78 classified into *Thermoleophilum* were resistant to radiation and are found primarily in extreme environment (Yakimov et al., 2003). Therefore, the enriched taxa and replacing taxa, which showed plant growth-promoting effects, largely led to the functional frame of rhizosphere bacterial community. Interestingly, regular changes in relative abundance of species with close correlations with crop growth are almost present in IDT-40 and IDT-50. It meant that IDT practice promoted crop growth through regulating rhizosphere bacterial community when the depth reached 40 cm.

The effects of different tillage practices on rhizosphere bacterial communities were reflected in the different functional profiles of the reassembled communities. Considering the close relationship of the rhizosphere bacterial community and plant growth (Gu et al., 2019), this may suggest that the distinctive soil-dependent microbial community were quite different in their functional patterns. Consistent with rhizosphere bacterial community structure, the functional structure was quite different when IDT depth reached 40 cm compared with CK, and there was no significant difference between IDT-40 and IDT-50. A previous study found that the most important functions of rhizosphere bacterial community were to improve host stress tolerance (Ren et al., 2020). Although plants recruit different microorganisms to support their functional requirements as they grow in soil, these microbes might belong to different taxa due to different filtering effects from environments (Ren et al., 2020). Thus, the species replacement processes of rare taxon caused by deep tillage had fewer effects on the presence/absence of function genes observed in rhizosphere soil, but significantly affected their abundances. With increasing tillage depth, more functions associated with signal transduction and biosynthesis of other secondary metabolites were enriched, which could enhance the stress tolerance of the host plant. Some signal molecules released by rhizosphere microbes could induce the expression of defense proteins within the plant (Jain et al., 2020). Rhizosphere microbes may introduce prokaryotic defense system via some signal protein to protect the host plant from diverse pathogens (Singh et al., 2013). Thus, plants presented stronger resistance to pathogens and decreased the potential to be infected, especially in IDT-50. Consequently, it can be argued that deep tillage enriched for microbes was associated with stress tolerance while maintaining the original functional composition, so that plants could better cope with the environmental stress and enhance growth. However, the results might be a prediction based on PICRUSt, and more convincing results through metagenomic analysis and metabolome analysis were required to verify the conclusion.

## Conclusion

Our study clearly demonstrated that IDT practices broke the restriction of field soil degradation under long-term conventional tillage and facilitated crop growth. IDT depth also played important roles in shaping rhizosphere soil bacterial community, soil characters, and RSA. Three mechanisms on how IDT practices and its depth regulated crop growth were suggested: (1) IDT practices improved soil fertility and nutrient acquisition through the roots, and deeper IDT significantly increased RL. (2) IDT-40 and IDT-50 helped to enrich crop growth-promoting bacteria. (3) Functions associated with stress tolerance in rhizosphere bacterial communities were increased in IDT-40 and IDT-50. Thus, the application of IDT practices in agriculture is a potential approach to increase plant physiological growth status and resistance to diseases, and 50 cm might be an appropriate IDT depth. However, additional investigations with IDT practices in various agricultural regions are required to verify the accuracy of the optimal IDT depth at 50 cm.

## Data Availability Statement

The datasets presented in this study can be found in online repositories. The names of the repository/repositories and accession number(s) can be found below: https://www.ncbi.nlm.nih.gov/, PRJNA692565.

## Author Contributions

YL, ZZ, JuL, HY, and YG designed the study, created the figures, and wrote the manuscript. MC, PC, GT, SD, ZW, and MZ performed the experimental work, sampling, and DNA sequencing. DM, JX, and JiL carried out the bioinformatics and statistical analysis. All authors helped in editing and completing the manuscript.

## Conflict of Interest

The authors declare that the research was conducted in the absence of any commercial or financial relationships that could be construed as a potential conflict of interest.

## Publisher’s Note

All claims expressed in this article are solely those of the authors and do not necessarily represent those of their affiliated organizations, or those of the publisher, the editors and the reviewers. Any product that may be evaluated in this article, or claim that may be made by its manufacturer, is not guaranteed or endorsed by the publisher.

## Footnotes

1
http://ieg4.rccc.ou.edu/index.cgi

2
http://129.15.40.240/MENA/main.cgi

3
https://github.com/picrust/picrust2/
