# Supplementary material for: Mechanism of Intermittent Deep Tillage and Different Depths Improving Crop Growth From the Perspective of Rhizosphere Soil Nutrients, Root System Architectures, Bacterial Communities, and Functional Profiles
Source: Front Microbiol. 2022 Jan 10;12:759374. doi: 10.3389/fmicb.2021.759374 (PMC8784561; doi:10.3389/fmicb.2021.759374)
Supplement: Supplementary file 1 [file Data_Sheet_1.docx]

**Mechanism of intermittent deep tillage and different depths improving crop growth from the perspective of rhizosphere soil nutrients, root system architectures, bacterial communities and functional profiles**

Yabing Gu^1^, **Yongjun Liu^23*^**, Jiaying Li^4^, Mingfeng Cao^5^, Zhenhua Wang^6^, Juan Li^2^, Delong Meng^1^, Peijian Cao^8^, Shuhui Duan^3^, Mingfa Zhang^7^, Ge Tan^a^, Jing Xiong^a^, Huaqun Yin^a^, **Zhicheng Zhou^3*^**

^1^ School of Minerals Processing and Bioengineering, Central South University, Changsha, 410083, China

^2^ College of Agronomy, Hunan Agricultural University, Changsha, China

^3^ Tobacco Research Institute of Hunan Province, Changsha, China

^4^ Yongzhou Tobacco Company of Hunan Province, Yongzhou, China

^5^ Changde Tobacco Company of Hunan Province, Changde, China

^6^ Zhangjiajie Tobacco Company of Hunan Province, Zhangjiajie, China

^7^ Xiangxizhou Tobacco Company of Hunan Province, Jishou, China

^8^ China Tobacco Gene Research Center, Zhengzhou Tobacco Research Institute of CNTC, Zhengzhou, China

* Corresponding author:

Yongjun Liu, [Vincentliu2020@163.com](mailto:Vincentliu2020@163.com), Tel: +008618374992422;

Zhicheng Zhou, [zhichengzhou_cs@sina.com](mailto:zhichengzhou_cs@sina.com), Tel: +008613507458555.

E-mail:

Yabing Gu: [guyabing0207@163.com](mailto:guyabing0207@163.com);

Yongjun Liu: [Vincentliu2020@163.com](mailto:Vincentliu2020@163.com);

Jiaying Li: [jiaying-li217@139.com](mailto:jiaying-li217@139.com);

Mingfeng Cao: 50511580@qq.com;

Zhenhua Wang: 11815401@qq.com;

Juan Li: adalee619@163.com;

Delong Meng: delong.meng@csu.edu.cn;

Peijian Cao: peijiancao@163.com;

Shuhui Duan: [285234028@qq.com](mailto:285234028@qq.com);

Mingfa Zhang: [zhangmingfa98@163.com](mailto:zhangmingfa98@163.com);

Ge Tan: tange_csu@csu.edu.cn;

Jing Xiong: [195612128@csu.edu.cn](mailto:195612128@csu.edu.cn);

Huaqun Yin: yinhuaqun_cs@sina.com;

Zhicheng Zhou: [zhichengzhou_cs@sina.com](mailto:zhichengzhou_cs@sina.com).


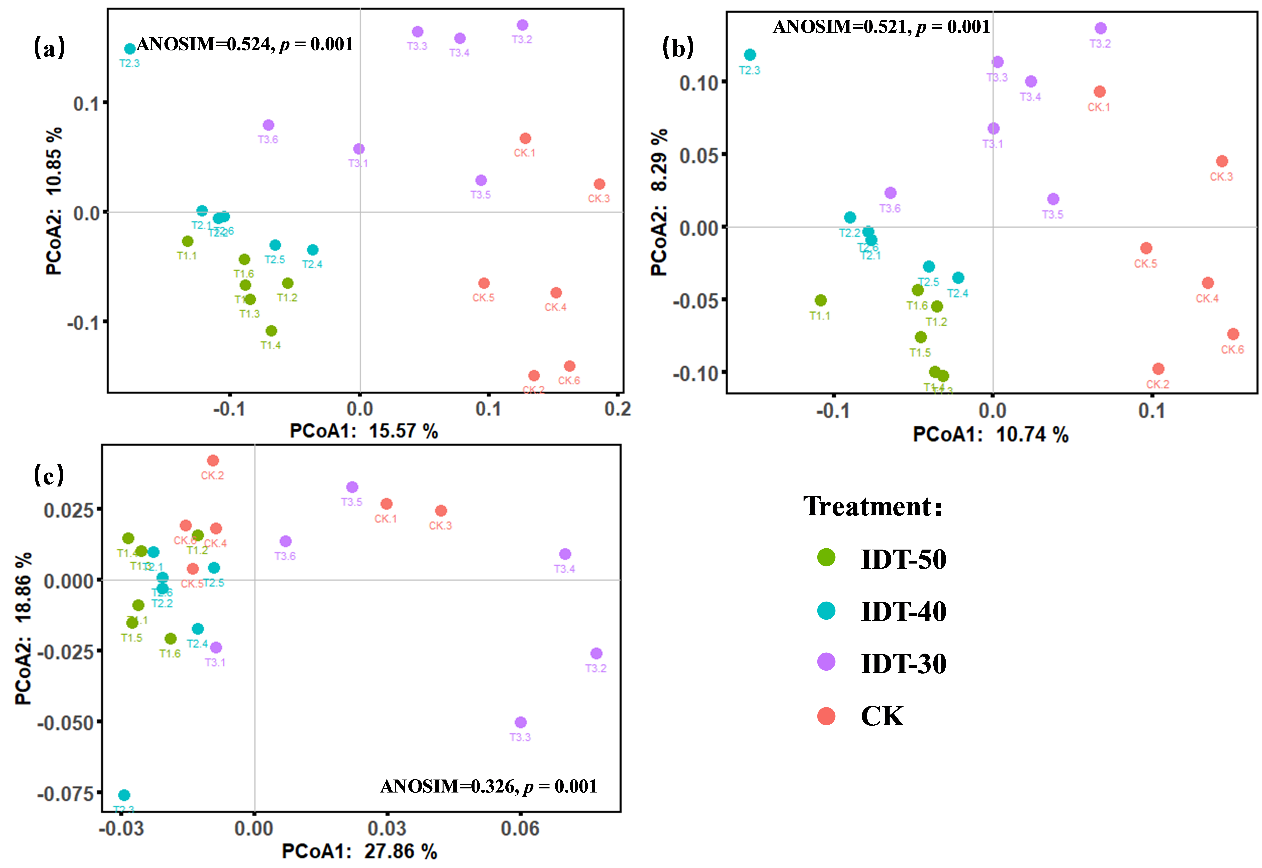


Figure. S1 Principal coordinates analysis (PCoA) revealing the difference of rhizosphere bacterial community from the four treatments based on Bray–Curtis dissimilarity matrix (a), unweighted UniFrac distance (b), and weighted UniFrac distance (c).


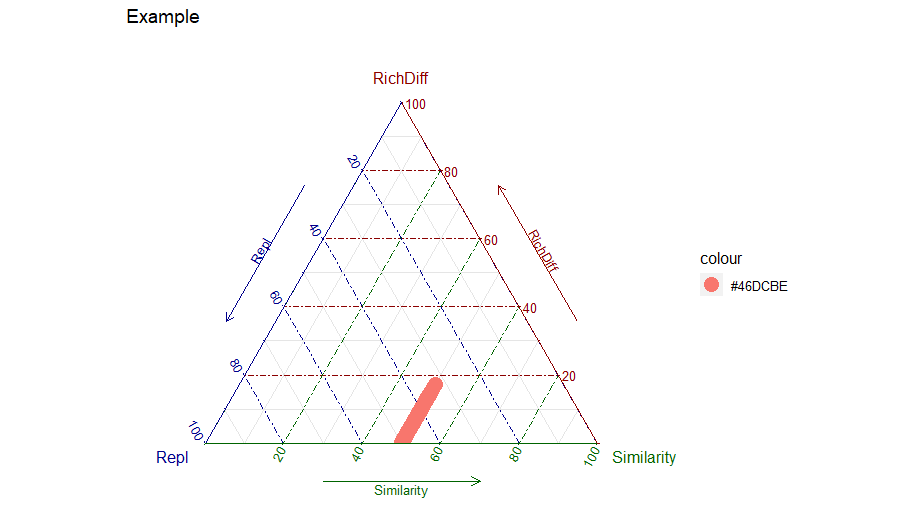


**Figure. S2** Triangular plots of beta diversity comparisons (using Jaccard dissimilarity index) for bacterial communities among all treatments. Each point represents a pair of sites. Its position is determined by a triplet of values from the S (similarity), Repl (replacement) and RichDiff (richness difference) matrices; each triplet sums to 100. The mean values of S, Repl and RichDiff are shown.


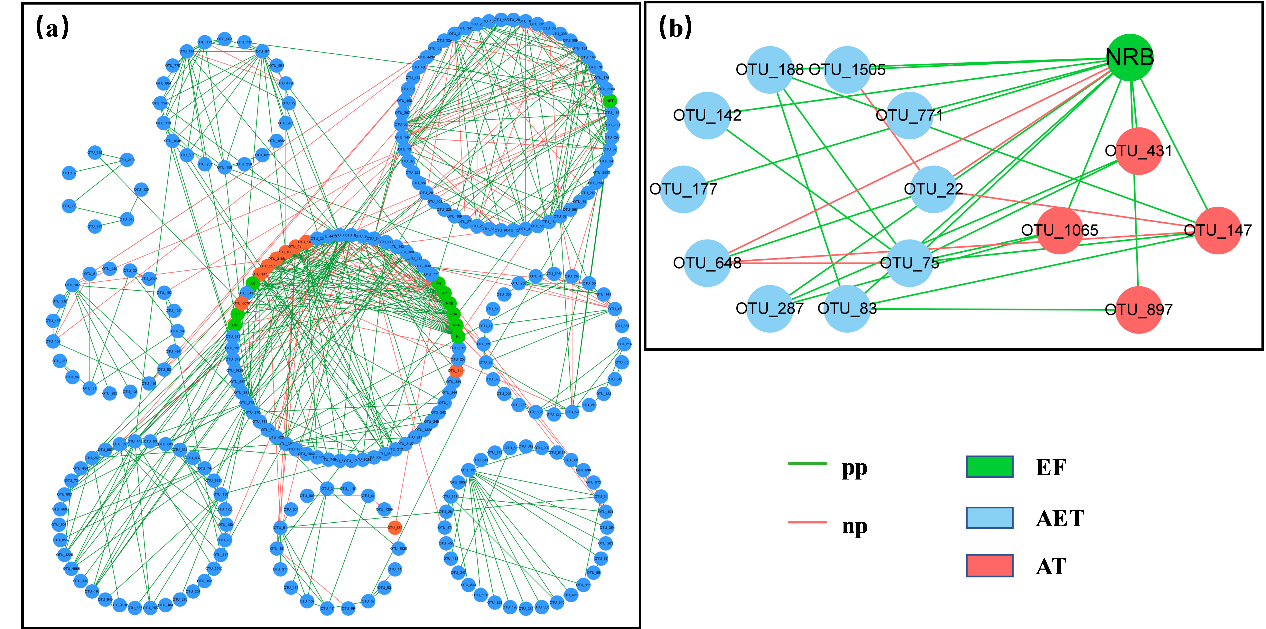


**Figure. S3** Radom matrix theory (RMT)-based molecular ecology networks (MENs). (a) Overall network. (b) Subnetwork interactions of NRB. The interactions of NRB with OTUs in AT and AET. Blue lines represented the interactions between nodes (OTUs) were negative and red lines represented positive interactions. EF: rhizosphere environmental variations; AET: OTUs shared in four treatments, AT: special OTUs present in deep tillage treatments.

**
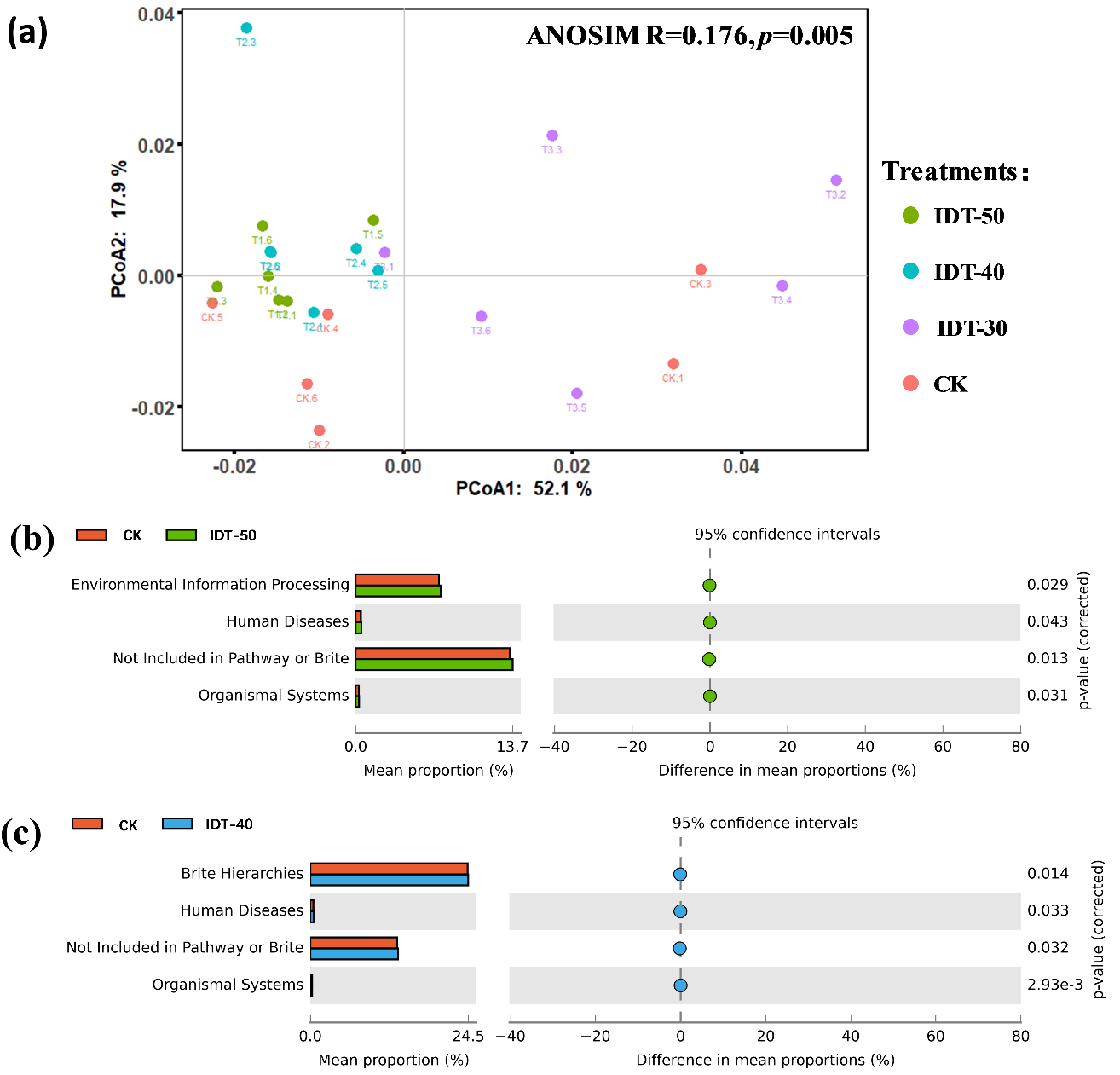
**

**Figure. S4** PICRUSt predicted metagenome functions. (a) Principal coordinates analysis (PCoA) revealing the difference of rhizosphere bacterial functional profiles from four treatments based on Bray–Curtis dissimilarity matrix. (b) Post-hoc plot showing KEGG level 1 function terms that differed significantly between T1 and CK. (c) Post-hoc plot showing KEGG level 1 function terms that differed significantly between T2 and CK.

**Table S1.** The alpha diversity of rhizosphere bacterial community. Values are presented as mean ± SD (n = 6), different letters indicate significant difference by one-way ANOVAs (Turkey's test at p < 0.05).

| Sample | chao value | Simpson Index | Shannon Index | Species richness | Pielou's evenness |
| --- | --- | --- | --- | --- | --- |
| IDT-50 | 2852.7±135.5a | 0.995±0.0011a | 6.474±0.0859a | 1848.5±63.8a | 0.861±0.0084a |
| IDT-40 | 2740.7±223.7ab | 0.993±0.0036a | 6.348±0.2280a | 1779.2±149.3ab | 0.848±0.0211a |
| IDT-30 | 2473.5±144.4b | 0.990±0.0056a | 6.105±0.3329a | 1611.7±118.3b | 0.827±0.0377a |
| CK | 2688.5±232.8ab | 0.995±0.0019a | 6.410±0.2066a | 1755.8±142.4ab | 0.858±0.0184a |

**Table. S2** Topological properties of the network.

| **Network Indexes** |  |
| --- | --- |
| Total nodes | 347 |
| Total links | 647 |
| R square of power-law | 0.906 |
| Average degree (avgK) | 3.729 |
| Average clustering coefficient (avgCC) | 0.16 |
| Average path distance (GD) | 4.97 |
| Geodesic efficiency (E) | 0.246 |
| Harmonic geodesic distance (HD) | 4.07 |
| Maximal degree | 25 |
| Nodes with max degree | OTU_75 |
| Centralization of degree (CD) | 0.062 |
| Maximal betweenness | 6161.165 |
| Nodes with max betweenness | OTU_165 |
| Centralization of betweenness (CB) | 0.097 |
| Maximal stress centrality | 45537 |
| Nodes with max stress centrality | OTU_165 |
| Centralization of stress centrality (CS) | 0.718 |
| Maximal eigenvector centrality | 0.308 |
| Nodes with max eigenvector centrality | RL |
| Centralization of eigenvector centrality (CE) | 0.283 |
| Density (D) | 0.011 |
| Reciprocity | 1 |
| Transitivity (Trans) | 0.268 |
| Connectedness (Con) | 0.606 |
| Efficiency | 0.986 |
| Hierarchy | 0 |

**Table. S3** Relative abundance and classifier of top 35 taxa for distinguishing between tillage treatments. Relative abundance (%) is mean value of treatment. Blue values mean significant enriched OTUs compared to conventional tillage treatment (*p*<0.05), and red values mean significant decreased OTUs (*p*<0.05).

| **OTU** | **Phylum** | **Genus** | **IDT-50** | **IDT-40** | **IDT-30** | **CK** |
| --- | --- | --- | --- | --- | --- | --- |
| OTU_160 | Actinobacteria | Thermoleophilum | **0.048** | **0.057** | 0.218 | 0.207 |
| OTU_165 | Actinobacteria | Conexibacter | **0.028** | **0.045** | 0.128 | 0.147 |
| OTU_244 | Actinobacteria | Unclassified | **0.007** | **0.010** | 0.045 | 0.072 |
| OTU_183 | Actinobacteria | Conexibacter | **0.033** | 0.038 | 0.130 | 0.102 |
| OTU_406 | Actinobacteria | Gaiella | **0.022** | 0.030 | 0.118 | 0.080 |
| OTU_296 | Actinobacteria | Thermoleophilum | **0.018** | **0.027** | 0.127 | 0.105 |
| OTU_36 | Actinobacteria | Unclassified | **0.095** | 0.153 | 0.373 | 0.293 |
| OTU_192 | Actinobacteria | Aquihabitans | **0.138** | **0.107** | 0.037 | 0.040 |
| OTU_78 | Actinobacteria | Thermoleophilum | **0.043** | 0.080 | 0.298 | 0.175 |
| OTU_216 | Actinobacteria | Gaiella | **0.038** | **0.025** | 0.092 | 0.100 |
| OTU_147 | Unclassified | Unclassified | **0.095** | **0.178** | 0.012 | 0.000 |
| OTU_647 | Candidatus Saccharibacteria | Saccharibacteria_genera  _incertae_sedis | **0.037** | **0.022** | 0.000 | 0.000 |
| OTU_22 | Proteobacteria | Bradyrhizobium | **0.352** | **0.302** | 0.532 | 0.627 |
| OTU_6024 | Actinobacteria | Streptomyces | **0.170** | **0.237** | 0.075 | 0.060 |
| OTU_717 | Proteobacteria | Kofleria | **0.075** | **0.078** | 0.038 | 0.020 |
| OTU_193 | Proteobacteria | Mizugakiibacter | **0.080** | **0.203** | 0.032 | 0.015 |
| OTU_293 | Actinobacteria | Gaiella | **0.043** | **0.048** | 0.113 | 0.153 |
| OTU_62 | Actinobacteria | Unclassified | **0.123** | **0.123** | 0.285 | 0.293 |
| OTU_177 | Unclassified | Unclassified | **0.107** | **0.115** | 0.022 | 0.008 |
| OTU_483 | Proteobacteria | Aquabacterium | 0.228 | **0.278** | 0.105 | 0.123 |
| OTU_142 | Candidatus Saccharibacteria | Saccharibacteria_genera  _incertae_sedis | **0.085** | **0.185** | 0.022 | 0.005 |
| OTU_75 | Proteobacteria | Unclassified | **0.348** | **0.388** | **0.158** | 0.063 |
| OTU_143 | Acidobacteria | Gp6 | **0.542** | **0.397** | 0.210 | 0.128 |
| OTU_10 | Actinobacteria | Unclassified | **0.262** | 0.353 | 1.338 | 0.688 |
| OTU_593 | Acidobacteria | Gp3 | **0.038** | **0.023** | 0.005 | 0.000 |
| OTU_648 | Chloroflexi | Sphaerobacter | **0.000** | **0.003** | 0.018 | 0.037 |
| OTU_444 | Unclassified | Unclassified | 0.017 | **0.048** | 0.000 | 0.000 |
| OTU_1201 | Acidobacteria | Gp6 | 0.140 | 0.147 | 0.053 | 0.080 |
| OTU_6639 | Proteobacteria | Unclassified | **0.040** | **0.057** | 0.008 | 0.007 |
| OTU_188 | Gemmatimonadetes | Gemmatimonas | **0.108** | **0.238** | 0.028 | 0.003 |
| OTU_260 | Unclassified | Unclassified | **0.155** | **0.140** | 0.038 | 0.012 |
| OTU_71 | Gemmatimonadetes | Gemmatimonas | **0.267** | **0.377** | 0.163 | 0.117 |
| OTU_85 | Actinobacteria | Aeromicrobium | **0.605** | **0.560** | 0.280 | 0.258 |
| OTU_431 | Acidobacteria | Gp6 | **0.037** | **0.088** | 0.025 | 0.000 |
| OTU_2204 | Proteobacteria | Hydrogenophaga | **0.047** | 0.025 | 0.002 | 0.003 |
